# Automatic Speech Recognition and Large Language Models for Multilingual Pathology Report Generation: Proof-of-Concept Study

**DOI:** 10.2196/90814

**Published:** 2026-05-13

**Authors:** Kuan-Hsun Lin, Chia-Ping Chang, Chen-Tsung Kuo, Chien-Yeh Hsu, Shih-Hsin Hung, Chung-Yueh Lien, Siang Hao Lee, Yi-Chen Yeh, Yuan-Chia Chu

**Affiliations:** 1 Department of Information Management, Taipei Veterans General Hospital, No. 201, Sec. 2, Shipai Road, Beitou District, Taipei, 112201, Taiwan, +886 986-680623; 2 Department of Information Management, National Taipei University of Nursing and Health Sciences, Taipei, Taiwan; 3 Department of Pathology and Laboratory Medicine, Taipei Veterans General Hospital, Taipei, Taiwan; 4 Master Program in Global Health and Health Security, College of Public Health, Taipei Medical University, Taipei, Taiwan; 5 Department of Nursing, Taipei Veterans General Hospital, Taipei, Taiwan; 6 Department of Nursing, Chang Jung Christian University, Tainan, Taiwan; 7 School of Medicine, National Yang Ming Chiao Tung University, Taipei, Taiwan; 8 Big Data Center, Taipei Veterans General Hospital, Taipei, Taiwan

**Keywords:** speech recognition software, natural language processing, electronic health records, multilingual, pathology

## Abstract

**Background:**

Accurate transcription of pathology gross examination dictation is important for clinical documentation, but multilingual dictation remains challenging in settings where clinicians mix Chinese and English while final pathology reports are written in English.

**Objective:**

This study aimed to evaluate whether a Whisper-based automatic speech recognition (ASR) pipeline guided by contextual system messages and combined with open-source large language models (LLMs; Qwen2:72b, Llama3.1:70b, Gemma2:27b) could improve multilingual (Chinese-English) pathology dictation transcription accuracy and generate clinically appropriate English gross description reports.

**Methods:**

We conducted a controlled proof-of-concept study using 125 simulated mixed Chinese-English pathology gross examination audio recordings created by physicians or pathologists. Audio recordings were transcribed using Whisper ASR with and without a contextual system message. The ASR transcripts were then converted into English gross description reports using 3 open-source LLMs: Qwen2:72b, Llama3.1:70b, and Gemma2:27b. Outcomes included character error rate, Bilingual Evaluation Understudy, Recall-Oriented Understudy for Gisting Evaluation (ROUGE)-1, ROUGE-2, ROUGE-L, Metric for Evaluation of Translation with Explicit Ordering, pathologist Win-Tie-Lose rankings, report-level error categories, inference time, and interrater agreement.

**Results:**

The ASR contextual system message reduced the mean character error rate from 0.344 (SD 0.176; 95% CI 0.313‐0.375) to 0.066 (SD 0.100; 95% CI 0.048‐0.084; *P*<.001). Qwen2:72b achieved the highest automated metric scores, including a Bilingual Evaluation Understudy of 0.644 (SD 0.307), ROUGE-1 of 0.866 (SD 0.163), ROUGE-2 of 0.771 (SD 0.235), ROUGE-L of 0.842 (SD 0.178), and Metric for Evaluation of Translation with Explicit Ordering of 0.805 (SD 0.214). Pathologist-coded total error rates were 16.8% (21/125) for Qwen2:72b, 45.6% (57/125) for Llama3.1:70b, and 92.8% (116/125) for Gemma2:27b. The exact agreement between the 2 pathologists across full ranking categories was 76.8% (96/125; Cohen κ=0.668), and agreement on the top-ranked model or tied top group was 81.6% (102/125; Cohen κ=0.722).

**Conclusions:**

In this proof-of-concept evaluation, contextual prompting improved ASR transcription accuracy, and Qwen2:72b generated the most accurate English pathology reports among the evaluated LLMs. However, the study used simulated audio recordings, a local vocabulary prompt, and report-level rather than term-level clinical annotation. LLM-generated reports should therefore be considered draft documentation requiring pathologist verification, and prospective validation in real clinical workflows is needed before clinical deployment.

## Introduction

Automatic speech recognition (ASR) technologies are increasingly being implemented in health care to improve workflow efficiency by automating the transcription of spoken input into text [1,2]. Accurate transcription of medical records is fundamental to patient care and clinical decision-making. However, the intricate language used in medical settings, including technical terms, abbreviations, and context-specific expressions, presents significant barriers to achieving high transcription accuracy [3,4]. These limitations are particularly problematic in high-stakes environments such as pathology, where documentation errors can result in delays or incorrect diagnoses [5,6]. Errors in transcription can lead to misinterpretations, impacting patient safety and clinical outcomes.

Despite advancements in ASR technologies, transcription accuracy remains a challenge, particularly when dealing with specialized medical terminology and complex clinical workflows [7-9]. Current ASR systems often fall short, resulting in transcription errors that compromise the quality and reliability of electronic health records [10-12]. Therefore, improving the accuracy and reliability of medical transcription systems is imperative for enhancing clinical documentation and ensuring patient safety [13-15]. Moreover, directly using spoken language transcriptions in medical documents may be inappropriate, as clinicians’ speech often includes filler words, abbreviations, or incomplete pronunciations for the sake of efficiency. Converting spoken language transcriptions into the appropriate structured format remains a significant challenge. This challenge is even greater in multilingual settings. For example, in Taiwan, health care professionals frequently mix Chinese and English in spoken communication, while medical documents are typically written in English. As a result, directly using spoken language transcriptions in medical documents is not feasible.

Recent advancements in large language models (LLMs) offer a promising solution to these challenges. LLMs, with their sophisticated contextual understanding and ability to process vast amounts of data, have demonstrated the potential to enhance ASR outputs by reducing transcription errors and improving language comprehension in specialized domains [16,17]. While there is growing evidence supporting the use of LLMs in natural language processing, their application in medical transcription, especially in combination with ASR systems, remains underexplored. This study addresses this gap by integrating the Whisper ASR system with LLMs, including Qwen2:72b [18], Llama3.1:70b [19,20], and Gemma2:27b [21], to improve transcription accuracy and generate clinically appropriate pathology reports.

A key aspect in this study is the use of system messages—predefined instructions designed to guide the LLMs in accurately interpreting and generating clinical documentation [22]. These messages are tailored to address the challenges of medical transcription, such as recognizing specialized terms and avoiding irrelevant content. By leveraging system messages, the study aims to enhance the accuracy of the Whisper ASR system, particularly in environments requiring high levels of precision, such as the documentation of gross pathology findings.

This study, conducted in Taiwan, focuses on the gross examination of pathology specimens, a process where precision in documenting specimen characteristics is essential. Traditionally, this documentation has been manual, which can be both time consuming and prone to errors. By integrating artificial intelligence–powered ASR technology into the workflow, the process can be streamlined, allowing medical personnel to verbally dictate their findings while handling specimens [23]. This approach not only improves workflow efficiency but also reduces the potential for transcription errors, ultimately contributing to better clinical outcomes.

This study aimed to evaluate, in a controlled proof-of-concept setting, whether a Whisper-based ASR pipeline guided by system messages and combined with open-source LLMs could improve multilingual pathology dictation transcription and generate clinically appropriate English gross description reports. We hypothesized that system messages would reduce ASR transcription error and that LLM performance would differ across models in report quality, error profile, and inference time.

## Methods

### Study Design and Setting

**Figure 1.**
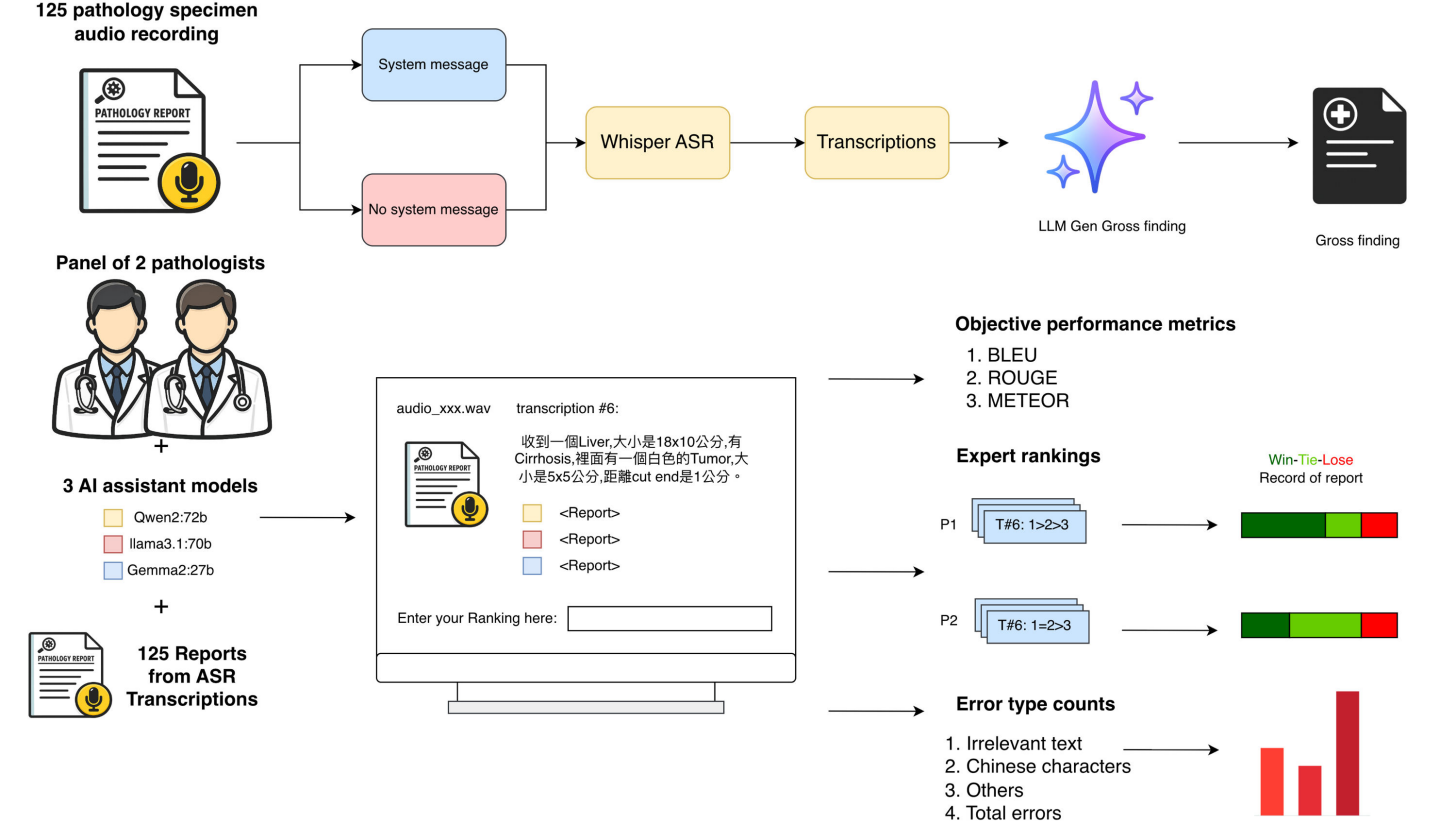
Study workflow diagram. This figure provides an overview of the controlled proof-of-concept study design, including simulated audio creation, automatic speech recognition (ASR) transcription with and without contextual system messages, large language model (LLM)–based English report generation, and evaluation by automated metrics and pathologist review. AI: artificial intelligence; BLEU: Bilingual Evaluation Understudy; ROUGE: Recall-Oriented Understudy for Gisting Evaluation; METEOR: Metric for Evaluation of Translation with Explicit Ordering.

This proof-of-concept study evaluated a hybrid ASR-LLM pipeline for multilingual pathology gross description documentation (Figure 1). The study used 125 simulated mixed Chinese-English audio recordings created by physicians or pathologists to reflect common gross examination dictation patterns in Taiwan. The recordings were not clinical recordings and did not contain real patient voices, patient data, or identifiable information. Each audio file was transcribed by Whisper ASR with and without a contextual system message. The resulting mixed-language transcripts were subsequently converted into English pathology gross description reports by 3 LLMs. Two pathologists evaluated the LLM-generated reports for clinical appropriateness and coded report-level errors. The study was designed as a controlled formative evaluation of technical feasibility and report quality rather than a clinical effectiveness or deployment trial.

### Model Selection and System Integration

#### Whisper ASR System

We selected the Whisper ASR system for its superior capability in transcribing multilingual medical dialogues. Whisper was chosen for its zero-shot accuracy across diverse language contexts—a crucial feature in environments involving both Chinese and English medical terms [8]. To quantify its effectiveness, we compared transcription results using the character error rate (CER), both with and without the application of system messages. These system messages are preconfigured prompts designed to enhance transcription precision.

#### LLM Selection

The Whisper ASR system was integrated with LLMs through a Flask-based application programming interface (API). Three open-source LLMs were evaluated: Qwen2:72b, Llama3.1:70b, and Gemma2:27b. All models received the same ASR transcript and the same LLM system message for each case. The LLM stage transformed the mixed Chinese-English ASR transcript into a standardized English gross description report. The LLMs were instructed to normalize terminology and formatting but not to add information beyond the transcript. No model-specific fine-tuning was performed.

#### System Messages

Two types of system messages were used (Multimedia Appendix 1). For ASR, the system message was provided to the Whisper transcription service as a contextual prompt and vocabulary guide at the time of transcription. CER was calculated on the resulting ASR transcript before any LLM-based report generation. Therefore, the CER comparison reflects transcripts generated with versus without the ASR contextual prompt, rather than postprocessing by the downstream LLM. For LLM report generation, a separate system message instructed the models to convert the mixed-language transcript into an English pathology report, preserve only information present in the transcript, avoid unsupported interpretation, and exclude nonreport text.

#### Computational Environment

All inference tasks were performed using 2 NVIDIA A100 graphics processing units (40 GB each), using the Ollama platform for local model deployment. The models were run using Ollama default generation settings, including the default temperature of 0.8. Other generation hyperparameters were not exhaustively optimized or varied, and this is acknowledged as a reproducibility limitation.

### Validation Process

#### CER Analysis for Whisper ASR Transcription

The transcription accuracy of Whisper ASR was evaluated using CER. Physician- or pathologist-prepared ground truth transcripts were used as the reference standard. For each audio recording, CER was calculated by comparing the ASR transcript with the corresponding ground truth transcript. CER was measured separately for transcripts generated with and without the ASR contextual system message, before any LLM-based report generation.

#### Expert Ranking and Win-Tie-Lose Analysis for LLM-Generated Reports

Two pathologists independently evaluated the reports generated by Qwen2:72b, Llama3.1:70b, and Gemma2:27b for each transcription task. Rankings were based on overall accuracy and suitability for pathology gross description reporting. Ties were permitted when reports were judged clinically similar. The resulting rankings were analyzed using a Win-Tie-Lose framework. Interrater agreement was assessed using exact agreement and Cohen κ.

#### Error Type Analysis for LLM-Generated Reports

Physician- or pathologist-coded report-level errors were categorized as irrelevant text, Chinese character output, other factual or report errors, and total error. Irrelevant text referred to comments, instructions, or conversational content not belonging in a pathology report. Chinese character output indicated that Chinese characters remained in the English report. Other errors included clinically relevant inaccuracies such as incorrect organ names, measurements, margins, or descriptive statements. Total error indicated the presence of any of these error categories in a report. Error rates were summarized as counts, percentages, and 95% CIs.

#### Evaluation Using Bilingual Evaluation Understudy, Recall-Oriented Understudy for Gisting Evaluation, and Metric for Evaluation of Translation With Explicit Ordering Metrics

Automated report generation performance was evaluated using Bilingual Evaluation Understudy (BLEU), Recall-Oriented Understudy for Gisting Evaluation (ROUGE)-1, ROUGE-2, ROUGE-L, and Metric for Evaluation of Translation with Explicit Ordering (METEOR) scores, with physician-prepared reference report summaries as the ground truth. These metrics were used to provide reproducible overlap-based comparisons across LLMs. As overlap metrics do not fully capture clinical correctness, they were interpreted alongside pathologist rankings and report-level error analysis.

#### Statistical Analysis

Continuous metrics are summarized as mean, SD, median, range, and 95% CI. For CER and automated text generation metrics, 95% CIs around the mean were estimated using the 2-tailed *t* distribution across the 125 paired recordings or generated reports. As CER values with and without ASR system messages were obtained from the same audio recordings, paired analyses were used to compare transcription performance. LLM-generated reports were compared across models using paired tests and nonparametric tests where appropriate. Report-level error rates are presented as counts, percentages, and Wilson 95% CIs. Interrater agreement between the 2 pathologists was assessed using exact agreement and Cohen κ.

#### Inference Speed Measurement

To compare the inference speed of 3 LLMs—Gemma2:27b, Qwen2:72b, and Llama3.1:70b—in a clinical setting, we measured the inference times as a complete cycle from audio input through the ASR system to the LLM’s output. Each model was tested using 125 audio samples across 10 epochs to ensure consistent and reliable results. The measurements included the mean, median, and maximum inference times, as well as the 95% CIs for each model.

### Ethical Considerations

This study used only simulated audio recordings created for research and system evaluation purposes. The recordings did not include human participants, patient audio, patient data, or identifiable information. Therefore, ethics approval and informed consent were not required. Future studies using real clinical recordings or patient-related data would require appropriate institutional review and privacy safeguards.

## Results

### System Integration and Performance Analysis

#### Overview

The integration of the Whisper ASR system with LLMs was systematically evaluated for accuracy and clinical applicability (Figure 1). The evaluation included 3 main components: the transcription accuracy of the Whisper ASR system, the Win-Tie-Lose analysis of the pathology reports generated by the 3 LLMs, and the error type analysis of these reports. We analyzed the differences between the transcriptions generated by Whisper ASR and the ground truth transcriptions using the CER, comparing the effects of including or excluding system messages. Expert pathologists evaluated the pathology reports produced by the 3 LLMs—Qwen2:72b, Llama3.1:70b, and Gemma2:27b—and assigned rankings based on the accuracy and clinical relevance of the content. Additionally, a detailed error type analysis was conducted on the pathology reports generated by the LLMs, comparing differences among the models.

#### Transcription Accuracy of the Whisper ASR System

Table 1 presents the CER results for Whisper ASR with and without contextual system messages. Without system messages, the mean CER was 0.344 (SD 0.176; median 0.293; 95% CI 0.313‐0.375). With system messages, the mean CER decreased to 0.066 (SD 0.100; median 0.024; 95% CI 0.048‐0.084), representing a mean paired reduction of 0.278 (*P*<.001). On the basis of this improvement, transcripts generated with the ASR contextual system message were used as inputs for subsequent LLM report generation.

**Table 1.** Character error rate (CER) with and without an automatic speech recognition (ASR) system message (N=125).

ASR condition	CER, mean (SD; 95% CI)^a^	Median (IQR)	*P* value^b^
With ASR system message	0.066 (0.100; 0.048‐0.084)	0.024 (0.000‐0.538)	<.001
Without ASR system message	0.344 (0.176; 0.313‐0.375)	0.293 (0.137‐1.040)	Reference

a Character error rate values were calculated against physician- or pathologist-prepared ground truth transcripts.

b *P* value reflects a paired comparison because both conditions were evaluated on the same 125 recordings.

#### Objective Metric Evaluation Across LLMs

**Figure 2.**
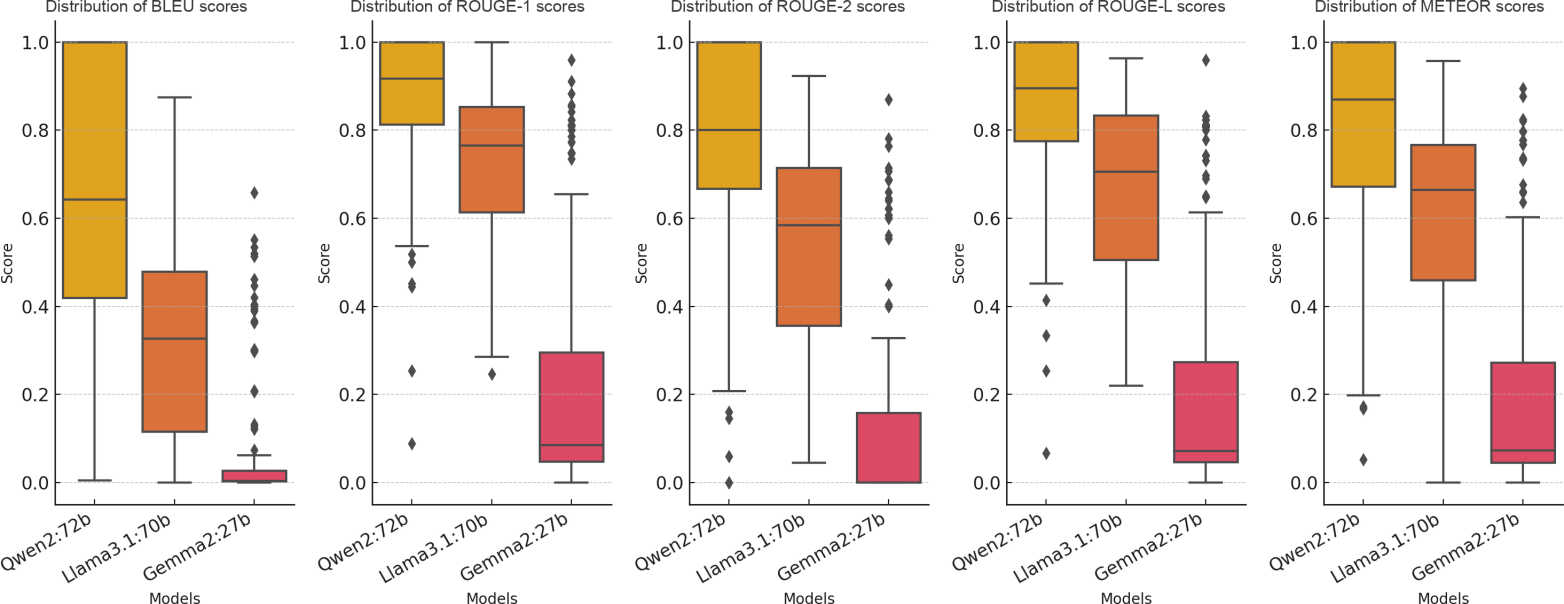
Distribution of Bilingual Evaluation Understudy (BLEU), Recall-Oriented Understudy for Gisting Evaluation (ROUGE), and Metric for Evaluation of Translation with Explicit Ordering (METEOR) scores across large language models. This figure presents the distributions of BLEU, ROUGE-1, ROUGE-2, ROUGE-L, and METEOR scores for Qwen2:72b, Llama3.1:70b, and Gemma2:27b. Full descriptive statistics are provided in Multimedia Appendix 1.

The performance of the LLM-generated pathology reports was assessed using BLEU, ROUGE-1, ROUGE-2, ROUGE-L, and METEOR scores, and the score distributions are shown in Figure 2. Full descriptive statistics are provided in Multimedia Appendix 1. Qwen2:72b achieved the highest scores across all metrics, with mean scores of 0.644 (SD 0.307; 95% CI 0.590‐0.699), 0.866 (SD 0.163; 95% CI 0.837‐0.895), 0.771 (SD 0.235; 95% CI 0.729‐0.813), 0.842 (SD 0.178; 95% CI 0.811‐0.874), and 0.805 (SD 0.214; 95% CI 0.767‐0.843) for BLEU, ROUGE-1, ROUGE-2, ROUGE-L, and METEOR, respectively. Pairwise Wilcoxon tests showed that Qwen2:72b outperformed Llama3.1:70b and Gemma2:27b across all 5 automated metrics (all *P*<.001).

Llama3.1:70b showed intermediate performance, with mean scores of 0.314, 0.717, 0.533, 0.675, and 0.604 for BLEU, ROUGE-1, ROUGE-2, ROUGE-L, and METEOR, respectively. Gemma2:27b had the lowest scores, with mean scores of 0.076, 0.227, 0.133, 0.203, and 0.203 for BLEU, ROUGE-1, ROUGE-2, ROUGE-L, and METEOR, respectively. Kruskal-Wallis tests across all 3 models were significant for all 5 metrics (all *P*<.001).

#### Win-Tie-Lose Distribution Across Models

**Figure 3.**
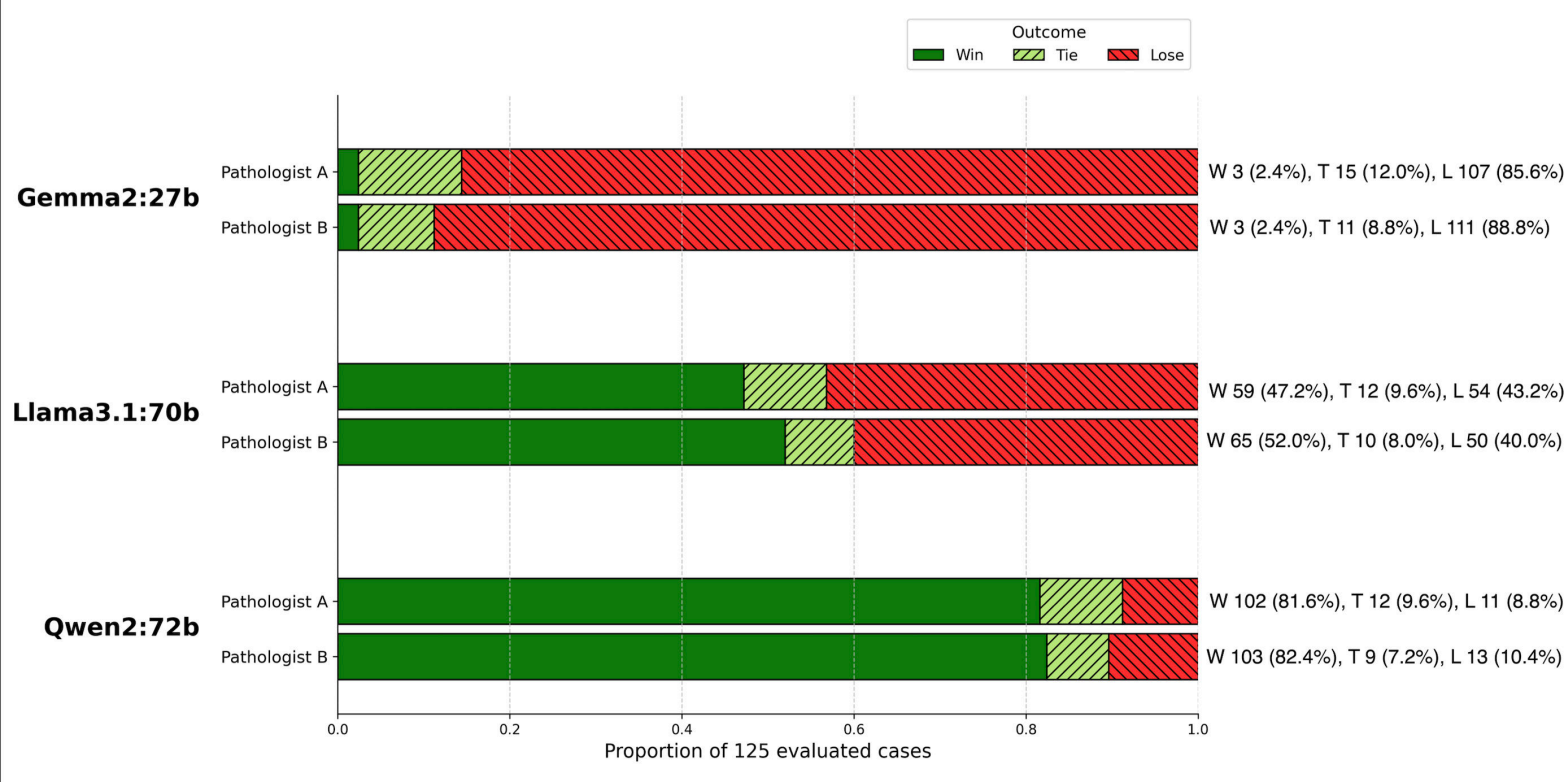
Model performance distribution: Win-Tie-Lose analysis. This figure illustrates the Win-Tie-Lose distribution for each large language model (Qwen2:72b, Llama3.1:70b, and Gemma2:27b) based on the 2 pathologists’ evaluations. L: lose; T: tie; W: win.

The Win-Tie-Lose framework was applied to assess the clinical relevance of outputs from the 3 LLMs (Figure 3). Qwen2:72b achieved the highest proportion of win classifications, whereas Gemma2:27b had the highest proportion of lose classifications. The 2 pathologists showed substantial agreement in the ranking assessment (Table 2): exact agreement across the full ranking categories was 76.8% (96/125), with an unweighted Cohen κ of 0.668. Agreement on the top-ranked model or tied top group was 81.6% (102/125), with a Cohen κ of 0.722.

**Table 2.** Interrater agreement for pathologist rankings (N=125).

Agreement analysis	Agreement, n (%)	Cohen κ	Interpretation
Full ranking category	96 (76.8)	0.668	Substantial agreement
Top-ranked model or tied top group	102 (81.6)	0.722	Substantial agreement

#### Error Type Distribution Across Models

**Figure 4.**
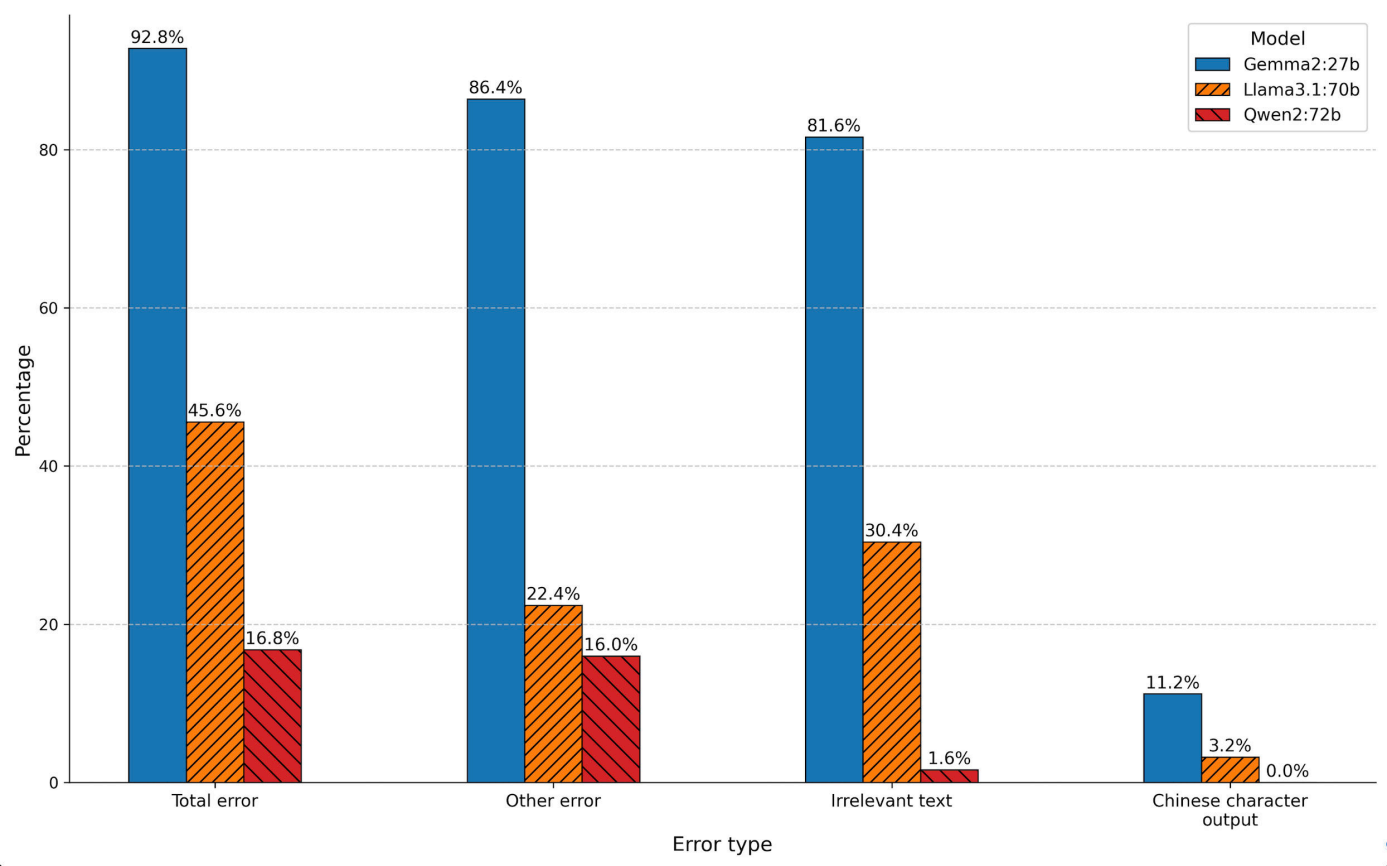
Error type distribution. This figure visualizes the distribution of pathologist-coded report-level error categories across the 3 large language models, including irrelevant text, Chinese character output, other factual or report errors, and total errors.

Error type analysis is shown in Figure 4 and summarized numerically in Table 3. Qwen2:72b had the lowest total error rate (21/125, 16.8%; 95% CI 11.3%‐24.3%), followed by Llama3.1:70b (57/125, 45.6%; 95% CI 37.1%‐54.3%) and Gemma2:27b (116/125, 92.8%; 95% CI 86.9%‐96.2%). These findings indicate that Qwen2:72b had the most favorable report-level error profile among the evaluated models.

**Table 3.** Pathologist-coded report-level error rates (N=125).

Model and error category		Values, n (%)	95% CI
Qwen2:72b			
Irrelevant text		2 (1.6)	0.4‐5.6
Chinese characters		0 (0)	0.0‐3.0
Other factual or report error		20 (16.0)	10.6‐23.4
Total error		21 (16.8)	11.3‐24.3
Llama3.1:70b			
Irrelevant text		38 (30.4)	23.0‐38.9
Chinese characters		4 (3.2)	1.3‐7.9
Other factual or report error		28 (22.4)	16.0‐30.5
Total error		57 (45.6)	37.1‐54.3
Gemma2:27b			
Irrelevant text		102 (81.6)	73.9‐87.4
Chinese characters		14 (11.2)	6.8‐17.9
Other factual or report error		108 (86.4)	79.3‐91.3
Total error		116 (92.8)	86.9‐96.2

### Impact of LLM Integration on Whisper ASR System for Pathology Report Generation

In Multimedia Appendix 1, we present examples of pathology reports generated by the 3 LLM models alongside their corresponding Whisper ASR transcriptions from the audio recordings. These examples illustrate both successful terminology correction and failure modes such as irrelevant text, Chinese character output, and incorrect factual or report content.

### Inference Speed Comparison

The mean inference times for the models were similar, ranging from 5.17 to 5.43 seconds (Multimedia Appendix 1). Qwen2:72b and Llama3.1:70b showed longer maximum inference times than Gemma2:27b, indicating that latency outliers should be considered in future real-time implementations.

## Discussion

### Principal Findings

This proof-of-concept study evaluated a hybrid ASR-LLM pipeline for multilingual pathology report generation. The main findings were that ASR contextual prompting substantially reduced CER, Qwen2:72b achieved the best automated text generation scores and the lowest pathologist-coded report-level error rate, and 2 pathologists showed substantial agreement in their model ranking assessments. These results support the technical feasibility of combining ASR and LLMs for multilingual pathology documentation, while also highlighting the need for human verification and real-world validation.

### Comparison With Prior Work

Prior systematic reviews of speech recognition for clinical documentation have found that speech recognition can reduce report turnaround time and support documentation efficiency, but evidence is heterogeneous and documentation errors remain a major concern [13]. Hodgson and Coiera [24] reported speech recognition accuracy ranging from 88.9% to 96.0% across included studies and emphasized the need to evaluate error types and clinical outcomes. Johnson et al [25] similarly noted that implementation depends on workflow, training, templates, accents, and system selection. Our study is consistent with this literature in showing improved transcription accuracy with contextual guidance but extends prior work by evaluating a multilingual pathology-specific ASR-LLM pipeline rather than ASR alone.

Few prior studies have specifically evaluated ASR for pathology gross examination dictation, and even fewer have examined mixed Chinese-English dictation followed by LLM-based English report generation. In another non-English medical context, Lee et al [26] compared cloud-based speech recognition APIs for Korean medical terminology using real physician-patient conversations and found medical term recognition accuracies of 75.1%, 50.9%, and 57.9% across 3 APIs. Compared with that study, our controlled simulation used a different evaluation setting and metric, achieving a low mean CER with ASR contextual prompting; however, Lee et al [26] used real clinical conversations and formal term-level medical terminology evaluation. This contrast highlights both the promise of contextual prompting in a pathology-specific pipeline and the need for future real-world validation with term-level concept annotation.

### Interpretation and Implications

The superior performance of Qwen2:72b suggests that model selection is important for multilingual medical documentation tasks. However, automated overlap metrics such as BLEU, ROUGE, and METEOR do not fully capture clinical correctness, unsupported inference, omission, or hallucination. The pathologist-coded error analysis therefore provides an important complement to automated metrics. Although LLMs sometimes corrected plausible ASR errors, this behavior may also introduce unsupported assumptions. Generated reports should therefore be treated as draft documentation requiring pathologist verification rather than autonomous clinical records [27].

The fixed vocabulary used in the ASR contextual prompt likely contributed to the CER improvement. This is clinically useful in a specialized setting such as pathology gross examination, where recurrent organ names, margins, measurements, and descriptive terms are common. At the same time, this design limits generalizability. Other hospitals, specialties, languages, accents, and staff dictation styles would require local vocabulary adaptation and prospective validation.

### Limitations

This study has several limitations. First, all audio recordings were simulated by physicians or pathologists in a controlled setting. Although this design enabled reproducible evaluation without patient data, it does not fully capture background noise, speaker variability, interruptions, overlapping speakers, or the operational complexity of real pathology grossing rooms. Second, only 2 pathologists evaluated the generated reports. We added interrater agreement statistics, but future studies should include more raters from multiple institutions. Third, the original annotation schema captured report-level errors but did not label every organ, measurement, margin, unit, or bilingual expression separately. Therefore, formal concept-level accuracy and critical-term preservation could not be calculated retrospectively without additional annotation. Fourth, LLM generation used local Ollama defaults, and not all hyperparameters were exhaustively optimized. Finally, this study did not evaluate integration with electronic health record systems, turnaround time in live clinical workflows, or downstream patient safety outcomes.

### Conclusions

This controlled proof-of-concept study suggests that contextual ASR prompting combined with LLM-based report generation may support multilingual pathology documentation. Qwen2:72b showed the strongest overall performance among the evaluated models, with the highest automated metric scores and the lowest report-level error rate. However, the findings should be interpreted as formative evidence rather than clinical validation. Future work should evaluate the pipeline prospectively in real pathology workflows, include broader speaker and environmental variability, perform term-level clinical concept annotation, and define human verification safeguards before clinical deployment.

## Supplementary material

10.2196/90814 Multimedia Appendix 1 Contextual system messages used for Whisper automatic speech recognition transcription and large language model–based pathology report generation.

## Abbreviations

API: application programming interface
ASR: automatic speech recognition
BLEU: Bilingual Evaluation Understudy
CER: character error rate
LLM: large language model
METEOR: Metric for Evaluation of Translation with Explicit Ordering
ROUGE: Recall-Oriented Understudy for Gisting Evaluation
