# Supplementary material for: Automatic Speech Recognition and Large Language Models for Multilingual Pathology Report Generation: Proof-of-Concept Study
Source: JMIR Form Res. 2026 May 13;10:e90814. doi: 10.2196/90814 (PMC13170781; doi:10.2196/90814)
Supplement: Multimedia Appendix 1 [file formative-v10-e90814-s001.docx]

**Supplementary Materials**

Manuscript title: Automatic Speech Recognition and Large Language Models for Multilingual Pathology Report Generation: Proof-of-Concept Study

Manuscript ID: 90814

Journal: JMIR Formative Research

# Authors: Kuan-Hsun Lin, Chia-Ping Chang, Chen-Tsung Kuo, Chien-Yeh Hsu, Shih-Hsin Hung, Chung-Yueh Lien, Siang-Hao Lee, Yi-Chen Yeh, Yuan-Chia Chu

Manuscript #90814. This document contains supplementary materials supporting the revised manuscript.

## Supplementary File 1. Full System Messages for Whisper ASR and LLMs

System messages are reproduced from the original system-message table without shortening.

| **System Message Purpose** | **System Message (Original Language)** | **System Message (English Translation)** |
| --- | --- | --- |
| Whisper ASR System | 這是在臺灣的醫療場景，中英文混雜的口述病理標本描述情境，當中可能會有許多的醫學專有名詞和器官名稱。常見的英文專有名詞包括: colon, rectum, cecum, lung, gallbladder, cut margin, cut end, tumor, pleura, bronchus, liver, breast, skin, nodule, nevus, uterus, ovary, tube, myoma, endometrium, myometrium, vagina, margin, cut margin, cyst, proximal cut end, distal cut end, gastric cardia, fibrosis, lesion, submucosa, kidney, prostate, testis, ureter, seminal vesicle, urethra, necrosis, polyp, appendix。此外，也會有中文的器官名稱，例如: 胃, 食道, 大腸, 腎臟, 脾臟, 胰臟, 膀胱, 前列腺, 子宮。常見的描述文字例如: 檢體, 最大塊, 多塊, 收到, 距離。口述的發音可能不一定標準，也可能只有唸出字的一部份發音。如果遇到無法辨識的字，或是意義不合理的字請優先辨識為上面這些字。 | This is a medical scenario in Taiwan, where mixed-language pathology specimen descriptions (Chinese and English) are spoken, which may include many medical terminologies and organ names. Common English medical terms include: colon, rectum, cecum, lung, gallbladder, cut margin, cut end, tumor, pleura, bronchus, liver, breast, skin, nodule, nevus, uterus, ovary, tube, myoma, endometrium, myometrium, vagina, margin, cut margin, cyst, proximal cut end, distal cut end, gastric cardia, fibrosis, lesion, submucosa, kidney, prostate, testis, ureter, seminal vesicle, urethra, necrosis, polyp, appendix. There are also Chinese organ names, such as: stomach, esophagus, colon, kidney, spleen, pancreas, bladder, prostate, uterus. Common descriptive words include: specimen, largest piece, multiple pieces, received, distance. The pronunciation of the dictation may not always be accurate, and sometimes only part of the word may be pronounced. If any unrecognizable or unreasonable words are encountered, please prioritize recognizing them as the above terms. |
| LLM System | 這個AI是一位醫師助手，請將醫療情境的中英混雜對話內容進行編修與標點符號排版，逐字稿內容可能會有辨識錯誤，例如可能將醫學專有名詞誤辨識為其他字詞，或是把英文辨識成發音相似的中文字詞，或是把英文辨識成發音相近的其他英文字。請整理時將不合情境邏輯的詞句修正。請不要多加入在逐字稿內容中沒有提到的資訊。  常出現的醫學專有名詞如下: Gallbladder, lung, Cut margin, margin, pleura, capsule, liver, tumor, nodule, myoma, cyst, mass 多塊, 檢體  這個AI會將修正後的逐字稿轉換成合適的英文病理報告。病理報告內容請務必如實回報，不要自行增加或修改內容，不要把自己當作醫生來推測或補充資料，僅限於逐字稿中的資訊。請不要加入其他場景，例如醫生對話的形式。這個AI回答的內容只可以出現英文病理報告的內容，不可以出現任何評論或建議，或是不在病理報告內的文字 (例如 correction)。範例: The specimen received measures 20x15x10 cm and corresponds to a uterus with multiple myomas. The largest myoma measures 5x5x5 cm. The right ovary measures 3x3x2 cm, while the accompanying tube measures 1 cm in diameter. | This AI serves as an assistant to the doctor. Please edit and format the mixed-language (Chinese-English) dialogue content for medical scenarios. The transcript content may contain recognition errors, such as medical terms being misrecognized as other words, English words being recognized as similar-sounding Chinese characters, or English words being misrecognized as phonetically similar English words. When editing, please correct any words or phrases that are not contextually logical. Please do not add any information that was not mentioned in the transcript.  Commonly appearing medical terms include: Gallbladder, lung, Cut margin, margin, pleura, capsule, liver, tumor, nodule, myoma, cyst, mass 多塊, 檢體  This AI will convert the corrected transcript into an appropriate English pathology report. The pathology report content must be reported truthfully. Do not add or modify the content by yourself. Do not speculate or supplement information as if you are a doctor, but only reflect the information from the transcript. Do not include other scenarios, such as doctor dialogue. The content provided by this AI should only contain the English pathology report and should not include any comments or suggestions, or any text not within the pathology report (e.g., correction). Example: The specimen received measures 20x15x10 cm and corresponds to a uterus with multiple myomas. The largest myoma measures 5x5x5 cm. The right ovary measures 3x3x2 cm, while the accompanying tube measures 1 cm in diameter. |

## Supplementary File 2. Full Automated Metric Summary for LLM-Generated Reports

Automated text-generation metrics are summarized across the 125 generated reports for each model.

| **Model** | **Metric** | **N** | **Mean** | **SD** | **Median** | **95% CI** | **P value vs Qwen2** |
| --- | --- | --- | --- | --- | --- | --- | --- |
| Qwen2:72b | BLEU | 125 | 0.644 | 0.307 | 0.642 | 0.590-0.699 | Reference |
| Qwen2:72b | ROUGE-1 | 125 | 0.866 | 0.163 | 0.917 | 0.837-0.895 | Reference |
| Qwen2:72b | ROUGE-2 | 125 | 0.771 | 0.235 | 0.800 | 0.729-0.813 | Reference |
| Qwen2:72b | ROUGE-L | 125 | 0.842 | 0.178 | 0.896 | 0.811-0.874 | Reference |
| Qwen2:72b | METEOR | 125 | 0.805 | 0.214 | 0.869 | 0.767-0.843 | Reference |
| Llama3.1:70b | BLEU | 125 | 0.314 | 0.227 | 0.327 | 0.274-0.355 | <.001 |
| Llama3.1:70b | ROUGE-1 | 125 | 0.717 | 0.177 | 0.765 | 0.685-0.748 | <.001 |
| Llama3.1:70b | ROUGE-2 | 125 | 0.533 | 0.235 | 0.585 | 0.491-0.574 | <.001 |
| Llama3.1:70b | ROUGE-L | 125 | 0.675 | 0.187 | 0.706 | 0.642-0.708 | <.001 |
| Llama3.1:70b | METEOR | 125 | 0.604 | 0.219 | 0.664 | 0.566-0.643 | <.001 |
| Gemma2:27b | BLEU | 125 | 0.076 | 0.157 | 0.004 | 0.048-0.104 | <.001 |
| Gemma2:27b | ROUGE-1 | 125 | 0.227 | 0.291 | 0.084 | 0.175-0.278 | <.001 |
| Gemma2:27b | ROUGE-2 | 125 | 0.133 | 0.241 | 0 | 0.091-0.176 | <.001 |
| Gemma2:27b | ROUGE-L | 125 | 0.203 | 0.270 | 0.071 | 0.155-0.251 | <.001 |
| Gemma2:27b | METEOR | 125 | 0.203 | 0.263 | 0.073 | 0.157-0.250 | <.001 |

## Supplementary File 3. Examples of LLM-Generated Pathology Reports

Representative examples include the ground-truth transcription, Whisper ASR transcription, generated reports from the three LLMs, and pathologist comments/rankings when available.

| **No** | **Ground truth transcription** | **Whisper ASR transcription** | **Qwen_Report** | **Llama3.1:70b report** | **Gemma2:27b report** | **Ranking (Pathologist A)** | **Ranking (Pathologist B')** | **Comment** |
| --- | --- | --- | --- | --- | --- | --- | --- | --- |
| 1 | 收到一條colon,大小是18x10公分﹐裡面有一個tumor,大小是3x2公分﹐tumor距離proximal cut end是3公分﹐距離distal cut end是2公分﹐另外有一個append,大小是3x1公分﹐ | 收到一條colon,大小是18x10公分﹐裡面有一個tumor,大小是3x2公分﹐tumor的距離proximal cut end是3公分﹐距離distal cut end是2公分﹐另外有一個append,大小是3x1公分﹐ | The specimen consists of a colon measuring 18x10 cm. It contains a tumor measuring 3x2 cm, located 3 cm proximal to the cut end and 2 cm distal to the cut end. Additionally, an appendix measuring 3x1 cm is present. | The specimen received measures 18x10 cm and corresponds to a colon with a tumor. The tumor measures 3x2 cm and is located 3 cm from the proximal cut end and 2 cm from the distal cut end. An appendix, measuring 3x1 cm, is also present. | The specimen received is a colon measuring 18 x 10 cm. It contains a tumor measuring 3 x 2 cm. The tumor is located 3 cm from the proximal cut end and 2 cm from the distal cut end. An appendix measuring 3 x 1 cm is also included. | Q=L=G | Q=L=G | In this example, all three models correctly generated appropriate pathology reports. Notably, although the audio recording and transcription only mentioned 'append,' all three LLM models were able to correctly infer that this term should be 'appendix' and produced accurate report descriptions. |
| 2 | 檢體有多塊，合起來是0.8×0.5×0.3公分,最大是0.8×0.1×0.1公分。 | 檢體有多塊?合起來是0.8×0.5×0.3公分,最大是0.8×0.1×0.1公分。 | The specimen consists of multiple fragments aggregating to a size of 0.8x0.5x0.3 cm, with the largest fragment measuring 0.8x0.1x0.1 cm. | The specimen consists of multiple fragments, aggregating to a size of 0.8 x 0.5 x 0.3 cm. The largest fragment measures 0.8 x 0.1 x 0.1 cm. | Okay, I understand. Please provide me with the Chinese-English mixed dialogue transcript and I will edit it for grammar, punctuation, and medical terminology accuracy. I will then convert the edited transcript into an English pathology report based solely on the information provided. I will make sure to: * Correct any misidentified medical terms. * Ensure the logic of the sentences flows naturally. * Only include information present in the original dialogue. * Avoid adding any interpretations, diagnoses, or suggestions. I am ready when you are! | Q=L>G | Q=L>G | In this example, both Qwen2 and Llama3 generated correct and appropriate pathology reports. However, Gemma2 failed to generate any relevant pathology content and simply repeated the instructions from the system message. |
| 3 | lung, 然後這個是右上肺,大小是18x10x5, tumor是3x2x1公分,白色的,距離Bronchus是2公分,距離Pleura是0.1公分,中間有Necrosis。 | 右上肺,大小是18x10x5, tumor是3x2x1公分,白色的,距離Branchus是2公分,距離Prova是0.1公分,中間有Necrosis。 | The right upper lobe of the lung measures 18x10x5 cm. A tumor within measures 3x2x1 cm and is white in color. The distance from the bronchus is 2 cm, and it is 0.1 cm away from the pleura. There is evidence of necrosis present centrally. | Here is the revised and formatted text: The right upper lung measures 18x10x5 cm. A tumor measuring 3x2x1 cm is present, which is white in color. The distance from the bronchus to the tumor is 2 cm, and the distance from the pleura to the tumor is 0.1 cm. Focal necrosis is noted within the tumor. Please let me know if this meets your requirements or if further revisions are needed! | Pathology Report Specimen: Right upper lobe of lung Size: 18 x 10 x 5 cm Tumor: 3 x 2 x 1 cm, white in color. Distance from Branchus: 2 cm Distance from Pleura: 0.1 cm Microscopic Features: Necrosis present within the tumor. | Q>L>G | Q>L>G | In this example, Qwen2 generated a correct and appropriate report. While the content of the Llama3-generated report was generally correct, it unnecessarily added irrelevant sentences, such as 'Please let me know if this meets your requirements or if further revisions are needed!' Gemma2's report also included incorrect and irrelevant information, such as 'Microscopic Features:'. Additionally, it is noteworthy that although Whisper's transcription erroneously recorded 'pleura' as 'Prova,' all three LLM models were able to infer that the term should be 'pleura' and correctly presented it as 'pleura' in the pathology report. In Whisper's transcript, 'Bronchus' was also mistakenly recorded as 'Branchus'; both Qwen2 and Llama3 were able to correctly present it as 'Bronchus' in the pathology report, while Gemma2 still wrote it as 'Branchus'." |
| 4 | 35575, 左下肺,大小是5.5×4.1×1.1公分, 有一顆黑色的nodule, 0.5×0.3×0.2公分,距離margin 1.1公分, 距離pleura 0.2公分 | 35575 左下肺大小是5.5×4.1×1.1公分有一顆黑色的nodule 0.5×0.3×0.2公分距離margin 1.1公分 距離prova 0.2公分 | The left lower lung specimen measures 5.5 x 4.1 x 1.1 cm and contains a black nodule measuring 0.5 x 0.3 x 0.2 cm. The distance from the nodule to the margin is 1.1 cm, and the distance to the pleura is 0.2 cm. | 左下肺切片中發現一枚大小為5.5×4.1×1.1公分的樣本，內有黑色結節（nodule），其尺寸為0.5×0.3×0.2公分。該結節距離邊緣（margin）約1.1公分，距離胸膜（pleura）約0.2公分。 | The specimen received is the left lower lobe of the lung measuring 5.5 x 4.1 x 1.1 cm. There is a black nodule measuring 0.5 x 0.3 x 0.2 cm. The distance from the nodule to the cut margin is 1.1 cm and the distance to the pleura is 0.2 cm. | Q=G>L | Q=G>L | In this example, both Qwen2 and Gemma2 generated correct and appropriate pathology reports, whereas the report generated by Llama3 erroneously included descriptions in Chinese characters. (Note: The '35575' in the transcription is a simulated pathology specimen number.) |
| 5 | 檢體7塊, 合起來是0.9×0.5×0.2, 最大是0.5×0.3×0.2。 | 檢體7塊, 合起來是0.9×0.5×0.2, 最大是0.5×0.3×0.2。 | The specimen consists of seven pieces aggregating to a size of 0.9 x 0.5 x 0.2 cm, with the largest piece measuring 0.5 x 0.3 x 0.2 cm. | The specimen consists of 7 fragments, aggregating to 0.9 x 0.5 x 0.2 cm in dimension. The largest fragment measures 0.5 x 0.3 x 0.2 cm. | 好的，我會盡力按照您的指示將中英混雜的病理報告逐字稿進行編修和標點符號排版，並轉化成合適的英文病理報告。 請提供您需要我處理的逐字稿內容，我會盡快為您完成翻譯工作。 | Q=L>G | Q=L>G | In this example, both Qwen2 and Gemma2 generated correct and appropriate pathology reports, whereas Gemma2 failed to generate any relevant pathology content and simply repeated the instructions from the system message. Additionally, all responses were in Chinese. |
| 6 | 35476, 6塊,合起來是1.5×1.2×0.3,最大的是1.3×0.9×0.3。 | 3,5,4,7,6,6塊,合起來是1.5×1.2×0.3,最大的是1.3×0.9×0.3。 | The specimen received consists of six pieces, aggregating to measure 1.5 x 1.2 x 0.3 cm collectively. The largest piece measures 1.3 x 0.9 x 0.3 cm. | Seven nodules are received, aggregating 1.5x1.2x0.3 cm. The largest nodule measures 1.3x0.9x0.3 cm. | I understand. Please provide me with the Chinese-English dialogue you would like me to edit and format. I will carefully revise it, paying close attention to medical terminology accuracy, logical flow, and proper punctuation. My output will be a concise English pathology report based solely on the information present in the edited transcript. I will refrain from adding any interpretations, suggestions, or extraneous details. | Q>L>G | Q>L>G | In this example, Qwen2 correctly generated an appropriate pathology report. Llama3 erroneously recorded the number of tissue fragments as 'Seven' (the correct number should be 'Six'). Gemma2 did not generate any relevant content and only repeated the instructions from the system message. |
| 7 | 收到一個Uterus, 10x10x5公分,左邊的ovary是3x2公分,tube是2公分,右邊的ovary是1x1x1公分,tube是3公分。 | 收到一個Uterus, 10x10.5公分,左邊的ovary是3x2公分,tube是2公分,右邊的ovary是1x1x1公分,tube是3公分。 | The specimen received measures 10x10.5 cm and corresponds to a uterus. The left ovary measures 3x2 cm, while the accompanying tube measures 2 cm. The right ovary measures 1x1x1 cm, and its corresponding tube measures 3 cm. | The specimen received measures 10x10.5 cm and corresponds to a uterus. The left ovary measures 3x2 cm, with an accompanying tube measuring 2 cm in length. The right ovary measures 1x1x1 cm, with an accompanying tube measuring 3 cm in length. | Okay, I understand. Please provide the Chinese-English dialogue transcript and I will edit it for grammar, punctuation, and medical terminology accuracy, then convert it into a concise English pathology report based solely on the information provided in the dialogue. I will not add any interpretations, diagnoses, or opinions â€“ just the factual findings from the transcribed conversation. | Q=L>G | Q=L>G | In this example, both Qwen2 and Gemma2 generated correct and appropriate pathology reports, whereas Gemma2 failed to generate any relevant pathology content and simply repeated the instructions from the system message. |
| 8 | 36370 Gallbladder, 大小是6x2.6, 厚度是0.2, 裡面有黑色石頭0.3x0.2x0.1, 沒有polyp | 36370 Goldblader大小是6x2.6厚度是0.2裡面有黑色石頭0.3x0.2x0.1沒有polyp | The gallbladder, measuring 6 x 2.6 cm in size with a wall thickness of 0.2 cm, contains a black stone measuring 0.3 x 0.2 x 0.1 cm. No polyps are identified. | Here is the revised text: The gallbladder measures 6x2.6 cm with a wall thickness of 0.2 cm. A black stone, measuring 0.3x0.2x0.1 cm, is present within the lumen. No polyps are identified. Or, in a more formal pathology report format: Gallbladder: The gallbladder measures 6x2.6 cm with a wall thickness of 0.2 cm. A single black stone, measuring 0.3x0.2x0.1 cm, is present within the lumen. No polyps or other significant lesions are identified. | The specimen received is a gallbladder measuring 6 x 2.6 cm with a thickness of 0.2 cm. There are black stones measuring 0.3 x 0.2 x 0.1 cm present within the gallbladder. No polyps were identified. | Q=G>L | Q=G>L | In this example, both Qwen2 and Gemma2 generated correct and appropriate pathology reports, whereas Llama3 inserted unrelated text and suggestions, such as "Here is the revised text:" and "Or, in a more formal pathology report format:". Notably, although the Whisper transcription erroneously recorded "gallbladder" as "goldblader", all three LLM models were able to correctly infer that this term should be "gallbladder" and produced accurate report descriptions. |
| 9 | 37176, 兩塊, 都是點3乘點2乘點1。 | 37176, 兩塊, 都是點3乘點2乘點1。 | The specimen consists of two pieces, each measuring 0.3 x 0.2 x 0.1 cm. | Two fragments of tissue are received, each measuring 0.3 x 0.2 x 0.1 cm. | Okay, I understand. Please provide me with the Chinese-English dialogue transcript. I will edit it for grammar and punctuation, correct any misrecognized medical terms, and then convert the revised text into an accurate English pathology report. I will only include information present in the transcript and avoid making any assumptions or adding external details. My response will solely be the English pathology report based on your input. | Q=L>G | Q=L>G | In this example, we tested a more concise method of describing specimen size, such as pronouncing '0.3' as '.3'. Both Qwen2 and Llama3 understood this notation and generated correct and appropriate pathology reports, whereas Gemma2 failed to generate any relevant pathology content and simply repeated the instructions from the system message |
| 10 | 37076,兩塊, 都是 .1.1.1。 | 37076,兩塊, 都是 .1.1.1。 | Two specimens were received, both measuring 1.1 x 1.1 x 1.1 cm. | Two fragments of soft tissue, each measuring 0.1 cm in greatest dimension. | Okay, I understand. Please provide me with the Chinese-English mixed dialogue transcript, and I will edit it for grammar, punctuation, and logical consistency based on the medical terminology you provided. I will then generate an English pathology report reflecting only the information present in the edited transcript. For example, if the transcript says "The gallbladder have tumor", I will correct it to "The gallbladder has a tumor" and generate a pathology report stating something like: "Microscopic examination reveals a tumor within the gallbladder." I am ready when you are! Please paste the dialogue. | L>Q>G | L>Q>G | In this example, we tested a more concise method for describing specimen size. We pronounced '0.1' as '.1' and did not say 'times'. Llama3 correctly understood this notation and generated an relatively accurate pathology report. Qwen2 misunderstood the specimen size notation, recording '.1' as '1.1'. Gemma2 failed to generate any relevant pathology content and simply repeated the instructions from the system message |

## Supplementary File 4. Inference Speed Analysis of Large Language Models

| **Model** | **Mean Time (s)** | **SD Time (s)** | **Min Time (s)** | **Max Time (s)** | **Median Time (s)** | **99th Percentile Time (s)** | **95% CI Lower (s)** | **95% CI Upper (s)** |
| --- | --- | --- | --- | --- | --- | --- | --- | --- |
| gemma2:27b | 5.307 | 3.099 | 2.297 | 33.90 | 4.820 | 21.34 | 5.135 | 5.479 |
| qwen2:72b | 5.170 | 4.562 | 2.584 | 115.71 | 4.510 | 21.87 | 4.917 | 5.423 |
| llama3.1:70b | 5.431 | 4.793 | 2.262 | 113.35 | 4.808 | 24.53 | 5.165 | 5.697 |
